# Mapping transition of care for rare endocrine conditions: findings from a cross-sectional survey by the Endo-ERN ToC Working Group

**DOI:** 10.1530/EC-25-0798

**Published:** 2026-04-15

**Authors:** Francesco Carlomagno, Matteo Spaziani, Charlotte MW Gaasterland, Svetlana Lajic, Nicole Reisch, Uta Neumann, Corinna Grasemann, Eva Kassi, Graziamaria Ubertini, Felix Reschke, Pietro Maffei, Annemarie Verrijn Stuart, Susan M O’Connell, Claudia Giavoli, Danilo Fintini, Evangelia Charmandari, Nienke R Biermasz, Violeta Iotova, Hedi L Claahsen-van der Grinten, Kirstine Stochholm, Dana Craiu, Andreea Ioana Badea, Aglaia Kyrilli, Enora Le Roux, Sebastian Neggers, Ulla Döhnert, Andrea M Isidori

**Affiliations:** ^1^ Department of Experimental Medicine, ‘Sapienza’ University of Rome, Rome, Italy; ^2^ Endo-ERN Centre ‘Azienda Ospedaliero-Universitaria’ Policlinico Umberto I’, Rome, Italy; ^3^ Department of Theorethical and Applied Sciences, eCampus University, Novedrate, Italy; ^4^ Amsterdam UMC, University of Amsterdam, Emma Children’s Hospital, Amsterdam, The Netherlands; ^5^ Department of Women’s and Children’s Health, Karolinska Institutet, Department of Pediatric Endocrinology, Karolinska University Hospital, Stockholm, Sweden; ^6^ Medizinische Klinik IV, LMU Klinikum München, Munich, Germany; ^7^ Department for Paediatric Endocrinology and Diabetology, Center for Chronically Sick Children, Charité-Universitätsmedizin Berlin, Corporate Member of Freie Universität Berlin, Humboldt-Universität zu Berlin, Berlin Institute of Health, Berlin, Germany; ^8^ Department of Pediatrics, University Medical Center of the Johannes Gutenberg University Mainz, Mainz, Germany; ^9^ Center of Expertise for Rare Endocrine Diseases (ENDO-ERN accredited), General Hospital of Athens LAIKO, National and Kapodistrian University of Athens, Athens, Greece; ^10^ Endocrinology and Diabetology, Bambino Gesù Children’s Hospital, IRCCS, Rome, Italy; ^11^ Center for Pediatric Diabetology, Endocrinology, and Metabolism, Children’s Hospital AUF DER BULT, Hannover, Germany; ^12^ Clinica Medica 3 Unit, Department of Medicine, Padua University, Padua, Italy; ^13^ Department of Pediatric Endocrinology, Wilhelmina Children’s Hospital, University Medical Center Utrecht, Utrecht, The Netherlands; ^14^ Diabetes and Endocrinology, Children’s Health Ireland at Crumlin, Dublin, Ireland; ^15^ Endocrinology Unit, Fondazione IRCCS Cà Granda Ospedale Maggiore Policlinico, Milan, Italy; ^16^ Department of Clinical Sciences and Community Health, Dipartimento di Eccellenza 2023-2027, University of Milan, Milan, Italy; ^17^ Division of Endocrinology, Metabolism and Diabetes, First Department of Pediatrics, National and Kapodistrian University of Athens Medical School, ‘Aghia Sophia’ Children’s Hospital, Athens, Greece; ^18^ Department of Medicine, Division of Endocrinology, and Center for Endocrine Tumors Leiden, Leiden University Medical Center, Leiden, The Netherlands; ^19^ Department of Paediatrics, Medical University of Varna, UMHAT “Sv. Marina”, Varna, Bulgaria; ^20^ Amalia Children's Hospital, Radboud University Medical Centre, Radboudumc Expert Center Sex & Gender, Nijmegen, The Netherlands; ^21^ Department of Endocrinology, Aarhus University Hospital, Aarhus, Denmark; ^22^ Pediatric Neurology Discipline, Neuroscience Department, “Carol Davila” University of Medicine, Center of Expertise of Rare Pediatric Neurological Disorders, Al Obregia Clinical Hospital, Bucharest, Romania; ^23^ Department of Endocrinology, Hôpital Universitaire de Bruxelles (H.U.B.)-Hôpital Erasme, Brussels, Belgium; ^24^ AP-HP, Paris Cité University, Robert-Debré University Hospital, Clinical Epidemiology Unit, INSERM, Paris, France; ^25^ Department of Medicine, Endocrinology Section, Erasmus University Medical Center, Rotterdam, The Netherlands; ^26^ Division of Paediatric Endocrinology and Diabetes, Department of Paediatrics and Adolescent Medicine, University Hospital Schleswig-Holstein, Lübeck, Germany

**Keywords:** transition, transitional care, rare endocrine conditions (RECs), patient centred care, transition of care working group (ToC WG), Endo-ERN, rare endocrine diseases

## Abstract

**Background:**

Adolescents and young adults with rare endocrine conditions face significant challenges during the transition from paediatric to adult healthcare systems. Despite increasing awareness, unstructured transitional care is frequent across Europe and is linked to adverse health outcomes, reduced adherence, and loss to follow-up.

**Objective:**

This study aimed to map existing models of care and identify key barriers and needs that could inform the development of standardised tools and recommendations to support improved transition processes within the European Reference Network on Rare Endocrine Conditions (Endo-ERN) framework.

**Methods:**

A cross-sectional, web-based survey was developed and disseminated by the ‘Transition of Care’ Working Group. The questionnaire comprised 31 items across 10 thematic domains and targeted both Endo-ERN and non-affiliated centres. Responses were collected between January and March 2025. Statistical and qualitative thematic analyses were performed.

**Results:**

A total of 111 responses were analysed from 80 centres across 21 European countries. Findings revealed marked heterogeneity in transition models, use of protocols, and availability of psychological support. Only about half of the centres reported shared paediatric–adult visits, and over one-third lacked follow-up strategies. E-health tools were underutilised despite expressed interest. A significant proportion of participants reported limited access to transition coordinators and heterogeneous privacy and data protection practices, highlighting concerns regarding General Data Protection Regulation (GDPR) compliance.

**Conclusion:**

The study underscores the need for standardised, patient-centred models of transitional care for rare endocrine conditions across Europe. Findings will inform the creation of harmonised tools and protocols to guide multidisciplinary collaboration and improve long-term outcomes.

**Plain language summary:**

Young people with rare endocrine conditions often struggle when moving from child to adult healthcare. We surveyed centres across Europe and found large differences in how this process is organised. Our findings highlight the need for clearer plans, better coordination, and tools to support a safer and more consistent transition for all patients.

## Introduction

Adolescents and young adults (AYAs) often face significant challenges when transitioning from paediatric to adult-oriented healthcare systems. This period coincides with complex developmental milestones – emotional, social, educational, and psychosexual – during which individuals with chronic endocrine disorders require personalised, continuous medical, psychological, and social support (1). These challenges are particularly relevant for AYAs affected by rare endocrine conditions (RECs), a heterogeneous group of disorders that fall within the European Commission definition of rare diseases, affecting no more than 5 in 10,000 individuals (European Commission, Rare Diseases, 2024 – http://data.europa.eu/eli/dec/1999/1295/oj). Due to their low prevalence, patients with RECs frequently encounter difficulties in accessing specialised care and maintaining continuity of care across the life course.

A major concern during this period is the increased risk of non-adherence to treatment and loss to follow-up, particularly among individuals with childhood-onset endocrine disorders (2). Discontinuity of care has been associated with poorer adherence to treatment and adverse long-term outcomes, including increased morbidity and mortality (3). Additional challenges include the limited availability of adult specialists with expertise in rare childhood-onset disorders, suboptimal coordination between paediatric and adult services, and the reduced availability of multidisciplinary care compared with paediatric settings (4, 5).

Although general recommendations for transition in chronic conditions exist (NICE. Transition from children’s to adults’ services for young people using health or social care services, 2016, https://www.nice.org.uk/guidance/ng43), their implementation for rare endocrine disorders remains fragmented across Europe. The absence of widely adopted protocols, dedicated transition pathways, or structured services often results in heterogeneous practices driven by local resources and individual initiatives (6). Furthermore, transitional care for RECs frequently lacks dedicated processes, trained personnel, and tools to monitor long-term outcomes. Despite growing attention to this topic, important research gaps remain, particularly concerning how to define and measure successful transition. Although a consensus-based definition has been proposed (7), it does not provide operational thresholds for outcome evaluation, and no universally adopted standard currently exists, limiting comparability across programmes.

In parallel, the European Commission has established the European Reference Networks (ERNs) framework to improve care for patients with rare and complex conditions by facilitating cross-border collaboration and knowledge sharing (8). The network dedicated to RECs, Endo-ERN, currently includes more than 100 Reference Centres (RCs) across 27 European countries and is organised into eight Main Thematic Groups (MTGs), covering the major domains of endocrinology and promoting collaboration between paediatric and adult specialists.

Within this framework, the Endo-ERN Transition of Care Working Group (ToC WG) was established to improve transitional care for AYAs with RECs. The group brings together multidisciplinary expertise from all MTGs, including paediatric and adult endocrinologists, clinical researchers, and patient representatives, with the aim of developing practical tools, identifying barriers, and promoting best practices. Ongoing activities include workshops (most recently the 8th TALENT Conference Meeting held in Rome in February 2026), consensus initiatives, and educational resources to support clinicians and patients during transition.

As a first step towards these objectives, the ToC WG conducted a Europe-wide survey among healthcare professionals (HCPs) within Endo-ERN to assess current organisational models, clinical practices, and perceived challenges in transitional care for RECs (9). The primary aim of the present study was to describe the current landscape of transition practices across Europe, identify variability and gaps in care, and generate evidence to support the development of harmonised strategies and practical tools, including condition-specific checklists, to support clinical practice.

## Materials and methods

### Study design and objectives

This cross-sectional study was designed to explore current transitional care practices, organisational models, barriers, and perceived needs related to the transition of care for AYAs with RECs across Europe. It was initiated and coordinated by the ToC WG of Endo-ERN, whose members contributed to survey design, domain selection, pilot testing, and dissemination across participating centres. The primary objective of the study was to describe and analyse current transitional care practices in Europe. Secondary objectives were to identify key organisational barriers and unmet needs, and to gather insights to inform the development of practical tools and recommendations to improve transition processes.

### Survey development and distribution

A web-based questionnaire was developed using the Survio® platform (https://www.survio.com/), with careful attention to clarity, accessibility, and applicability across both paediatric and adult healthcare settings. Survey development and the selection of survey domains were iterative and informed by:

- a review of existing literature and clinical guidelines relevant to the transition of care for rare and chronic endocrine conditions,
- feedback from ToC WG members representing both paediatric and adult endocrinology, as well as clinical psychologists and patient advocates (e-PAGs), and
- a preliminary internal pilot conducted in 20 selected RCs to refine question phrasing and format.

These sources were used to identify the key domains considered most relevant for describing organisational models, clinical processes, and patient-centred aspects of transition care. The final version of the survey consisted of 31 items, distributed across key thematic areas. It included multiple-choice questions, single-choice items, and open-ended fields, allowing both quantitative and qualitative data collection, where applicable.

The survey was disseminated via email to all Endo-ERN RCs and affiliated healthcare professionals involved in transitional care, with a specific invitation to paediatric endocrinologists, adult endocrinologists, clinical psychologists, and transition coordinators. Further dissemination to non-Endo-ERN RCs was obtained through the ESE and ESPE members mailing-lists. The data collection period lasted approximately nine weeks, from 10 January to 14 March 2025. During this period, reminder emails were circulated through Endo-ERN, ESE, and ESPE communication channels to maximise participation.

### Survey content and domains

The questionnaire was structured around ten thematic domains, designed to capture a comprehensive picture of the transition of care processes:

- **Setting**: organisation and characteristics of transition pathways, including the presence of shared outpatient clinics or age-specific transition clinics (10).
- **Tools**: availability and use of written protocols, transition guidelines, and digital/e-health solutions (11, 12).
- **Caregiver involvement**: strategies to engage and support caregivers during the transition process (13).
- **Timing and criteria for transition**: how and when transition is initiated, and which criteria (e.g. age, clinical status, and patient readiness) guide this decision (14).
- **Collaboration among professionals**: multidisciplinary interactions and communication between paediatric and adult teams (15).
- **Psychological support**: role of mental health professionals, including their involvement, pre- and post-transition (16, 17, 18).
- **Long-term monitoring**: availability of structured follow-up protocols and mechanisms to reduce loss to follow-up (19, 20).
- **Equity of access to services**: barriers due to geographic, economic, or systemic inequalities (21, 22).
- **Patient experience**: whether and how patients’ perspectives are collected and incorporated into service design (23, 24).
- **Privacy and data security**: use of secure communication and documentation systems to ensure safe information transfer between care settings.

The full questionnaire is provided as Supplementary material (see section on Supplementary materials given at the end of the article).

### Participants

A total of 113 responses were collected, representing HCPs from 80 unique centres across 21 European countries. Of these, 65 responses came from Endo-ERN RCs, resulting in a participation rate of 63.1% (65/103 RCs). Participants included physicians dealing with the transition of paediatric patients (*n* = 51), adult patients (*n* = 20), or both (*n* = 42). While a single response per RC was encouraged, multiple responses from the same centre were accepted, particularly when transition practices differed between departments or patient populations.

### Statistical analysis

Descriptive statistical analyses were performed to explore the distribution of responses across thematic areas, using proportions and frequency distributions. Free-text entries were analysed qualitatively using thematic grouping to identify emerging categories and additional insights. Comparative analysis was planned across the type of healthcare setting (paediatric vs adult vs shared) and institutional affiliation (Endo-ERN RCs vs non-Endo-ERN centres).

Data analysis was conducted using Microsoft Excel and IBM SPSS (version 30.0), employing chi-square or Fisher’s exact tests for the analysis of categorical variables.

## Results

### Demographics

In total, 113 responses were recorded, of which 2 were excluded from further analysis (leaving 111 responses for the final analysis). One participant was representing a centre from Malaysia, which was evaluated as outside of the scope of the research questions. The other participant withdrew their data from further analysis since this person was not involved in care of subjects affected by RECs. A country-level overview of participating centres, the number of responses per centre, their Endo-ERN affiliation, and a list of non-participating Endo-ERN RCs are shown in Supplementary Table 1. A total of 21 countries were represented in the data, of which Italy (with 28 responses from 18 centres) and the Netherlands (with 16 responses from 8 centres) were represented the most.

### Setting

Of the participants, 17 (15.3%) reported that they were taking care of adults only, 51 (45.9%) that they were responsible for children only, and 43 (38.7%) that they cared for both adults and children in their clinic.

On the question ‘Does your centre provide shared outpatient clinics for transitioning patients?’, 57 participants (51.4%) answered ‘Yes, either a joint clinic or visits between paediatric and adult units before transfer. The adult physician (or specialised nurse) comes to the paediatric unit, or vice versa’. However, 27 participants answered that patients are directly referred to the adult unit at a specific age (e.g. ages 16–18) (24.3%). Other answers were either ‘a dedicated clinic for chronic endocrine conditions in adolescent and transition age patients (e.g. between the ages of 16 and 24)’ (*n* = 11, 9.9%) or other responses. For this question, the responses were not different between the participants who saw either only children or adults or both groups (as analysed with chi-square test: *X*^2^ (9, *n* = 111) = 11.8, *P* = 0.22); however, joint clinics were significantly more frequent among Endo-ERN centres (55.8 vs 18.8%, *P* = 0.012).

**Figure 1:**
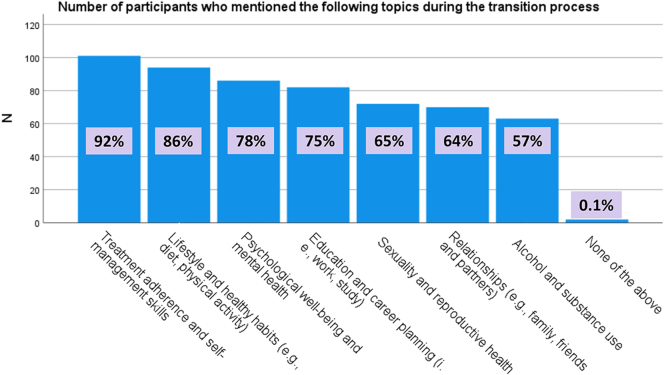
Number of participants who mentioned specific topics during the transition process of subjects affected by RECs.

In Fig. 1, we have depicted the number of participants who include the following topics in their transition process: lifestyle and healthy habits (e.g. diet and physical activity), alcohol and substance use, sexuality and reproductive health, relationships (e.g. family, friends, and partners), education and career planning (i.e. work and study), treatment adherence and self-management skills, psychological well-being and mental health, and none of the above. Other topics discussed by some participants during the transition process were driver’s license, patient’s history, and education about the condition and the transition period. HCPs involved in the transition of adult patients discussed significantly more topics than their paediatric counterparts (5.6 ± 1.8 vs 4.7 ± 2.2, *P* = 0.032).

### Tools

On the question of how many visits dedicated to the transition are conducted before the actual transfer, approximately half of the participants responded ‘1–2 visits’ (*n* = 58, 52.3%), 16 participants responded ‘none’ (*n* = 16, 14.4%), and some with ‘3 or more visits’ (*n* = 4, 3.6%). Sixteen participants responded that the number of visits varied, depending on the patient and condition (14.4%). For this question, the responses were not different between the participants who saw either only children or adults or both groups (as analysed with chi-square test: *X*^2^ (12, *n* = 111) = 15.5, *P* = 0.24).

On the question of whether guidelines for transition were used, the majority of participants responded that they used either locally developed protocols specific to the centre (*n* = 39, 35.1%) or nationally recognised protocols (*n* = 12, 10.8%) or condition-specific guidelines (*n* = 28, 25.2%). Approximately a quarter of participants responded that they used no standardised protocol at all (*n* = 27, 24.3%). There were no differences among Endo-ERN RCs and non-affiliated centres (*P* = 0.378).

The majority of the participants mentioned that they used questionnaires during the transition period (*n* = 69, 62.2%). The most mentioned reason for using standardised questionnaires was to identify a patient’s needs. E-health tools were not used by most of the participants (*n* = 71, 64.0%). The e-health tools that were mentioned as potentially being most beneficial for supporting transition in the future were mobile applications for patient education and monitoring (*n* = 42, 37.8%) and digital tools for patient self-management (such as reminders and trackers) (*n* = 22, 19.8%).

The two main barriers for implementing such e-health tools that were pointed out by the participants were a lack of resources or funding (*n* = 40, 36.0%) and a lack of standardised e-health platforms or guidelines (*n* = 29, 26.1%).

### Timing and criteria for transition

**Figure 2:**
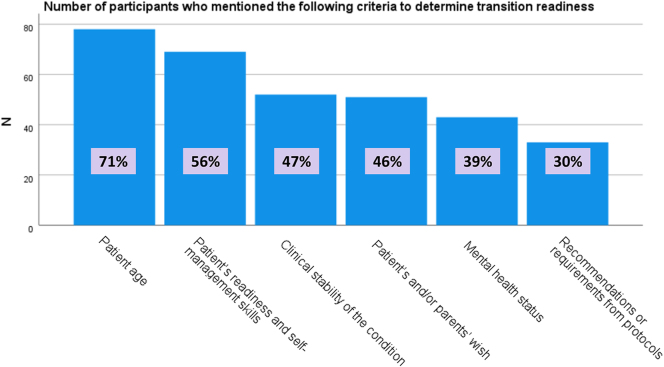
Number of participants who mentioned specific criteria to determine transition readiness for their patients.

The participants were asked what criteria they used, or considered appropriate, to determine the start of the transition process. In Fig. 2, the number of responses for the following criteria is depicted: patient age, clinical stability of the condition, patient’s readiness and self-management skills, mental health status, recommendations or requirements from local or national protocols, patient’s and/or parents’ wishes, or other.

The most common patient age group at which clinicians initiate the transition period was 16–18 years (*n* = 60, 54.1%), although some participants mentioned that this is highly individualised and that the initiation of this process should start earlier.

### Collaboration among professionals

To describe the main unmet needs in the collaboration between paediatric and adult physicians during transition, most participants answered ‘Co-creating individualised care plans that reflect the unique needs of transitioning patients’ (*n* = 42, 37.8%), ‘Establishing standardized protocols for transferring clinical information’ (*n* = 33, 29.7%), or ‘Ensuring effective communication of patient medical history, treatment plans, and ongoing needs’ (*n* = 18, 16.2%). Other given answers concerned mainly the lack of time, money, and resources. The key barrier to seamless collaboration between paediatricians and adult endocrinologists that was mentioned was a lack of shared outpatient clinics during the transition phase (*n* = 31, 27.9%), although some participants mentioned that the collaboration was already working well. The strategy to enhance the collaboration between paediatric and adult endocrinologists that was chosen most often by the participants was to organise joint discussions using standardised checklists or transition flowcharts (*n* = 46, 41.4%).

### Psychological evaluation and support

Most of the participants mentioned in the survey that the psychological readiness of their patients for the transition is not formally assessed (*n* = 63, 56.8%). For many of the participants, psychologists are not involved in transition planning (*n* = 32, 28.8%) or involved only for patients with specific psychological needs (*n* = 46, 41.4%). For some participants, there is no access to psychologists in their centre at all (*n* = 11, 9.9%). After the transition phase, the largest group of participants mentioned that responsibility is transferred to the psychologist in the adult care unit (*n* = 45, 40.5%), but many other participants mentioned that no psychological support is provided after the transition phase (*n* = 45, 40.5%).

### Long-term monitoring

On the question ‘How does your centre evaluate the effectiveness of long-term follow-up care post-transition?’, the largest group of participants responded that they did not evaluate long-term follow-up effectiveness (*n* = 41, 36.9%). However, another large group of participants answered that they were in regular communication with the adult centre’s personnel (*n* = 32, 28.8%). Challenges that were encountered to ensure long-term follow-up were mainly limited resources for ongoing monitoring (*n* = 29, 26.1%) and lack of dedicated transition coordinators (*n* = 29, 26.1%), although a substantial group of participants did not encounter any specific challenges (*n* = 25, 22.5%).

### Equity of access to services

**Figure 3:**
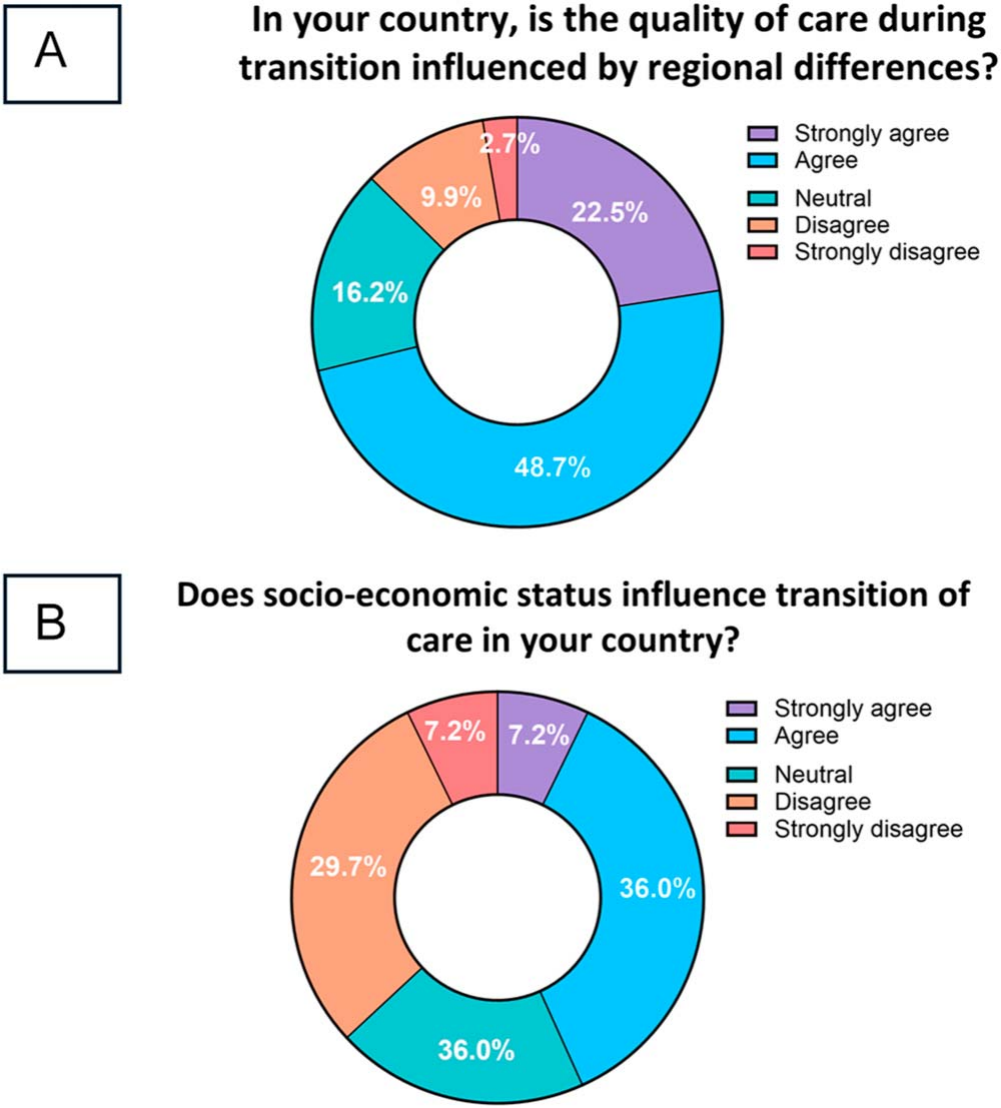
(A, B) Pie charts showing the participants’ perceived impact of regional (3A) and socio-economic (3B) differences on the quality of care during transition for individuals affected by RECs.

Participants were asked if they thought that regional differences were of influence on the quality of care during the transition process, and the majority of the participants agreed or strongly agreed with this statement (*n* = 79, 71.2%) (see Fig. 3). On the question of whether socio-economic status influences transition of care in their country, the opinions were more scattered (see Fig. 3).

### Patient experience

Most participants reported that in their centres, patient feedback on the transition process was either not collected systematically (*n* = 70, 63.1%) or collected only with regard to patients affected by specific conditions (*n* = 26, 23.4%). When feedback was collected, it was used mainly to identify gaps in the transition process, develop personalised care plans, or improve existing transition protocols.

### Privacy and data security

In the final questions of the survey, participants reported that they either explicitly obtain patient consent for medical data transfer (*n* = 30, 27.0%) or that consent is implicit in accepting patient transfer (*n* = 42, 37.8%) or that consent is not routinely obtained (*n* = 32, 28.8%) or that they were not aware of the consent procedures at their centres (*n* = 7, 6.3%). The patient’s medical history is transferred using either digital or secure platforms (*n* = 51, 45.9%) or paper-based records only (*n* = 18, 16.2%) or a combination of digital and paper records (*n* = 37, 33.3%).

## Discussion

In this survey, we gathered insights into the status of transitional care among endocrine HCPs throughout the EU, encompassing both Endo-ERN RCs and non-affiliated institutions. This report represents the preliminary outcome of the work conducted by the ToC WG, aimed at exploring current practices, challenges, and perceived needs in the transitional care of RECs.

The collected data clearly indicate significant variability in the quality of care and interventions among European countries, a finding consistent with previous literature reporting wide heterogeneity in transitional care models across healthcare systems (25, 26, 27). This is particularly evident, for example, in the divergent practices concerning the routine integration of psychological support and the systematic collection and use of patient feedback.

Recently, a joint ESE–ESPE clinical practice guidance has provided a comprehensive, consensus-based framework for transition of care in endocrine conditions, structured across key domains including care coordination, patient empowerment, multidisciplinary care, and psychological support (28). Our findings offer a complementary real-world perspective, highlighting the extent to which these recommended components are variably implemented across European centres.

Marked differences emerged not only between countries but also among individual centres. While a certain degree of heterogeneity is expected, and may reflect appropriate adaptations to local contexts and specific conditions, not all discrepancies can be regarded as adaptations of standardised transitional care pathways. Insufficient funding and the lack of dedicated care coordinators were frequently identified by participants as key barriers to providing consistent, high-quality transitional care, corroborating prior studies emphasising the critical role of care coordination and resource allocation in optimising transition outcomes (29). This issue may be particularly pronounced in countries where limited resources and infrastructural challenges further hinder the establishment of standardised transitional care pathways. Moreover, the observation that divergent responses were reported even within the same institution suggests a need for improved coordination at the intra-centre level (30, 31). Addressing these micro-level inconsistencies may represent a critical step towards achieving more equitable and standardised care pathways across the network. These variations may partly reflect the fact that, within the same hospital, different departments or MTG-related clinics may apply different approaches: some RCs may already have transition flowcharts in place, while others may lack such tools. These discrepancies highlight not only organisational challenges but also the lack of condition-specific transition guidelines. Although the recent ESE–ESPE guidance represents a major step forward in providing a structured and comprehensive framework (28), our data suggest that several of its core components are not yet consistently implemented in routine clinical practice across Europe. Tailored recommendations and practical tools for individual disorders could provide clinicians with clearer frameworks and help reduce the observed heterogeneity, thereby supporting the development of equitable and standardised care pathways across centres.

It should be noted that some variability in the responses may also reflect intrinsic methodological constraints, such as the use of mostly closed-ended items or the inherent difficulty of addressing all aspects of transitional care across different provider types. While not invalidating the results, these factors suggest a need for cautious interpretation. In addition, part of the observed variability likely stems from the absence of robust research evidence, shared guidelines, and a universally accepted definition of what constitutes a successful transition and how it should be achieved. In this context, it is not surprising that centres across Europe report highly divergent approaches to transition and transfer, even in highly specialised centres and by experienced HCPs.

Several concrete examples illustrate the lack of harmonisation across centres. While more than half of the participants reported organising shared outpatient visits between paediatric and adult services, a considerable number still rely on abrupt transfers based solely on age, without individualised care planning or structured joint consultations. Yet, combined consultations between paediatric and adult providers should be advocated as a best practice to ensure optimal transition, supporting continuity of care and reducing the risk of patient disengagement. Similarly, almost a quarter of participants indicated the absence of any formal protocol or guideline for managing transition, highlighting a critical gap in standardisation, and suggesting that transitional care is still not regarded as a systematic priority in many institutions.

In line with the ESE–ESPE guidance, which identifies the transition coordinator as a central figure in ensuring continuity of care (28), our findings highlight a substantial gap between recommendations and real-world availability of this role across centres. While this survey did not explore the topic in depth, transition coordinators are consistently recognised as the cornerstone of effective models, ensuring structured communication, individualised planning, and integration of patient perspectives. Their absence was frequently noted as a barrier, and prioritising the creation and funding of such roles should be considered a prerequisite for any sustainable improvement in the transition of care.

Similarly, while the joint ESE–ESPE guidance document strongly emphasises the integration of psychological assessment and support throughout the transition process (28), our results indicate that these services remain inconsistently available, suggesting a critical area for implementation efforts. Specifically, many centres reported limited access to key services such as initial psychological assessment, ongoing therapeutic support, or crisis intervention during and after transition. This is especially concerning in light of previous evidence showing that adolescents and young adults with RECs face increased emotional vulnerability, heightened stress, and risk of non-adherence during this period (16, 17). Moreover, more than one-third of centres acknowledged the absence of long-term follow-up strategies after transfer to adult care, an issue of particular importance in RECs, where ongoing monitoring is often essential to prevent complications and safeguard mental as well as physical health. These gaps may also reflect broader systemic limitations, including insufficient financial support within healthcare systems or restrictions related to insurance coverage. Taken together, these findings point to structural and process-based deficiencies in transition services, underscoring the urgent need for standardised protocols, dedicated coordination roles, and comprehensive psychosocial support to ensure continuity and quality of care.

Digital tools for self-management, education, and communication, although widely regarded as promising, remain underutilised in practice. Nonetheless, the strong interest in their future adoption represents an encouraging avenue for innovation. A recent scoping review identified a range of interventions, including online communication platforms and coaching strategies, that improved patient experiences during transitions, underscoring the potential of digital and personalised approaches to care planning (32). Equally promising is the reported use of structured questionnaires to assess patient needs, reflecting a growing awareness of the importance of tailored care strategies. Despite the increasing emphasis on patient data protection across the EU, the survey revealed considerable heterogeneity in consent procedures and data transfer practices during the transition process. While approximately one-third of participants reported obtaining explicit consent for the transfer of medical information, a comparable proportion either relied on implicit consent mechanisms or did not routinely obtain consent at all. Furthermore, a notable proportion of participants were unaware of the consent policies at their own centres.

These findings raise important concerns in light of the GDPR (Regulation EU 2016/679) (33), which designates health-related information as a special category of personal data requiring enhanced protection. According to Article 9 of the GDPR, the processing of such data must be based on explicit consent or other clearly defined legal grounds, and must ensure data minimisation, purpose limitation, and secure handling. The observed inconsistencies in both consent collection and methods of data transfer, ranging from secure digital platforms to paper-based systems, suggest a lack of harmonised protocols that could expose centres to ethical and legal vulnerabilities. This underscores the urgent need for Endo-ERN and affiliated institutions – together with scientific and professional societies such as ESPE, ESE, and national endocrine societies – to promote standardised, GDPR-compliant procedures to ensure the lawful, safe, and transparent management of patient data during transitional care. At the same time, it is equally important to guarantee that essential clinical information remains accessible across care settings, so that data protection does not inadvertently hinder continuity and quality of care.

Overall, the findings underscore the value of investing in care coordinators and the development of standardised, condition-specific tools to enhance consistency and cross-team collaboration. These results will inform the next steps of the ToC WG, supporting the co-design of harmonised, patient-centred models of care within Endo-ERN, together with clinicians, patients, and families. Future efforts should also aim to directly incorporate the perspectives of patients and caregivers, whose lived experiences may offer critical insights into the quality and continuity of transitional care. Importantly, beyond the responsibility of HCPs, governments and insurance systems also play a crucial role in recognising the special needs of patients with rare conditions and ensuring that appropriate resources are allocated to support effective transition.

### Strengths

This survey represents one of the first structured efforts to comprehensively assess transitional care practices for RECs across both Endo-ERN-affiliated and non-affiliated centres in Europe. One of its main strengths lies in its broad geographic coverage, with participation from 80 centres in 21 countries, providing a diverse and informative snapshot of current models of care.

The inclusion of both paediatric and adult endocrinologists allowed for a well-rounded perspective on the entire transition process, from early planning in paediatric settings to follow-up in adult care. The survey was collaboratively developed by clinicians within the ToC WG and underwent several rounds of refinement to ensure clarity and clinical relevance. While concise, it captured key domains such as care coordination, psychological support, the use of standardised tools, and digital health strategies, thereby integrating both clinical and organisational dimensions of transition.

The results of this study serve as a valuable foundation for identifying gaps and supporting the development of harmonised, patient-centred tools. Notably, the widespread interest among participants in adopting formalised protocols, structured needs assessments, and innovative digital solutions reflects a shared commitment to improving the quality and consistency of transitional care across Europe. These findings will help inform future Endo-ERN initiatives aimed at fostering more coordinated and responsive care pathways.

### Limitations

The study presents some limitations. First, although the survey was designed with input from both clinicians and patient representatives, it predominantly reflects the perspectives of HCPs. Some aspects, particularly those related to patient experience and the role of parents, may be better captured through the direct involvement of patients themselves, their families, or caregivers in future studies. This highlights an important area for future research, where patient input could provide a more comprehensive understanding of transitional care needs and experiences.

Second, the predominantly closed-ended design of the survey may have limited the ability to capture more nuanced or context-specific responses. While this format facilitated structured data collection across a large and heterogeneous sample, it may have constrained the expression of more individualised views.

Third, although both paediatric and adult clinicians contributed to the survey, some questions were not equally applicable to both professional groups. This may have affected the consistency and interpretability of some responses.

Finally, although participation from 80 centres across 21 countries is substantial, the sample may not fully represent the diversity of all Endo-ERN centres or other relevant stakeholders across Europe. Similarly, although the survey period was limited in duration, multiple reminders were distributed through several networks to optimise response rates. In addition, notwithstanding the overall number of responses and participating centres was substantial, the distribution across countries was uneven, with some countries contributing only a small number of centres. This prevented meaningful country-level comparisons and limited the interpretation of potential geographical differences in transition practices. Differences in response rates between countries or between larger and smaller centres may also have influenced the representativeness of the findings. Moreover, no single survey instrument can fully capture the complexity and variability of transitional care practices across such diverse clinical contexts and healthcare systems.

## Conclusion and future directions

This work represents an important first step in systematically describing the landscape of transitional care for RECs across Europe. The data will directly inform the development of practical tools, including standardised protocols and patient-centred checklists, by the Endo-ERN ToC WG. Importantly, our findings also provide empirical support for the priorities identified in the recent ESE–ESPE joint clinical guidance, while also highlighting key implementation gaps that should be addressed in future European strategies, explicitly addressing condition-specific needs, recognising the heterogeneity of rare endocrine conditions, and the importance of tailored approaches. Moving forward, further research involving patients, families, and national policymakers will be essential to validate these findings and co-create interventions – such as digital health tools, structured care pathways, and supportive policies – that are both scalable and responsive to the needs of this vulnerable population.

## Supplementary materials





## Declaration of interest

The authors declare that there is no conflict of interest that could be perceived as prejudicing the impartiality of the research reported.

## Funding

The research leading to these results has received funding from the European Union – NextGenerationEU through the Italian Ministry of University and Research under PNRR – M4C2-I1.3 Project PE_00000019 ‘HEAL ITALIA’ to AMI CUP B53C22004000006. The views and opinions expressed are those of the authors only and do not necessarily reflect those of the European Union or the European Commission. Neither the European Union nor the European Commission can be held responsible for them.

## Ethical approval and consent

This study did not involve human subjects or patient data. It consisted solely of an anonymised survey of healthcare professionals regarding organisational practices. According to institutional and EU regulations, ethical approval and informed consent were not required.
