# Supplementary material for: Mapping transition of care for rare endocrine conditions: findings from a cross-sectional survey by the Endo-ERN ToC Working Group
Source: Endocr Connect. 2026 Apr 15;15(4):e250798. doi: 10.1530/EC-25-0798 (PMC13097265; doi:10.1530/EC-25-0798)
Supplement: Supplementary file 1 [file supplementary_materials.pdf]

Endo-ERN 'Transition of care' survey for rare endocrine disorders

Dear Participant,

Thank you for taking the time to participate in this survey, developed by the **Endo-ERN 'Transition of Care' Working Group**.

This survey aims to explore current practices, challenges, and strategies in managing the **transition of care** for patients with **rare endocrine diseases** across different healthcare providers and institutions. The questions address key aspects of evaluation, treatment, follow-up, and multidisciplinary collaboration during the **transition from pediatric to adult care**.

Survey Details:

- Estimated completion time: **15-20 minutes**.

- All responses will be treated as **confidential** and will be used exclusively for research purposes within the framework of Endo-ERN.

At the end of the survey, you will also have the opportunity to share **comments** or **suggestions** to further enhance this research.

If you have any questions or need further clarification, please feel free to contact us at:

andrea.isidori@uniroma1.it & u.doehnert@uni-luebeck.de (Chairs)

francesco.carlomagno@uniroma1.it & matteo.spaziani@uniroma1.it (Managers)

Thank you for your valuable contribution!

Endo-ERN Transition of Care Working Group

1 Respondent's name, surname and e-mail address

Question instructions: *E.g. Dr. John Smith, john.smith@university.com*

2 Respondent's centre

Question instructions: *E.g., 'Azienda Ospedaliero-Universitaria Policlinico Umberto I' of Rome, Italy*

3 Is your centre...?

Question instructions: *Select one answer in each row*

	Yes	No
Involved in the transition of patients affected by endocrine diseases	◯	◯
Involved in the transition of patients affected by RARE endocrine diseases	◯	◯
An Endo-ERN centre	◯	◯

4 Are you...?

Question instructions: *Select one answer in each row*

	Yes	No
Responsible or involved in the transition of PAEDIATRIC patients?	◯	◯
Responsible or involved in the transition of ADULT patients?	◯	◯
An ESE member?	◯	◯
An ESPE member?	◯	◯

Setting

Evaluate current clinical setups (e.g., shared outpatient clinics, visit frequency) and standardized transition protocols from paediatrics to adult care.

5 Does your centre provide shared outpatient clinics for transitioning patients?

Question instructions: *Select one answer*

- ◯ a) Yes, a dedicated clinic for chronic endocrine conditions in adolescent and transition age patients (e.g., between the ages of 16 and 24)
- ◯ b) Yes, either a joint clinic or visits between paediatric and adult units before transfer. The adult physician (or specialised nurse) comes to the paediatric unit, or vice versa
- ◯ c) No, patients are directly referred to the adult unit at a specific age (e.g., ages 16-18)
- ◯ d) Other (please specify) ______

6 In case, how many joint visits are conducted before the actual transfer?

Question instructions: *Select one answer*

- ◯ a) None
- ◯ b) 1-2 visits
- ◯ c) 3 or more visits
- ◯ d) A variable number, depending on the individual patient/condition
- ◯ e) N/A, we do not provide joint clinics or visits

7 Do you use standardized clinical protocols or guidelines for transition and evaluation of transition efficiency at your clinic?

Question instructions: *Select one answer*

- ◯ a) No standardized protocols are used
- ◯ b) Locally developed protocols specific to the centre
- ◯ c) Nationally recognized protocols
- ◯ d) Condition-specific guidelines
- ◯ e) Other guidelines or recommendations (please specify) ______

8 Which of the following topics are routinely addressed during the transition process at your centre?

Question instructions: *Select all that apply*

- ☐ a) Lifestyle and healthy habits (e.g., diet, physical activity)
- ☐ b) Alcohol and substance use
- ☐ c) Sexuality and reproductive health
- ☐ d) Relationships (e.g., family, friends and partners)
- ☐ e) Education and career planning (i.e., work, study)
- ☐ f) Treatment adherence and self-management skills
- ☐ g) Psychological well-being and mental health
- ☐ h) None of the above
- ☐ i) Other (please specify) ______

Tools

Assess the use of questionnaires for psychological feedback, and e-Health tools supporting transition. Examine specific tools, such as 'transition

readiness' questionnaires, patient education courses and for monitoring patient progress.

9 What is the primary purpose of using questionnaires in the transition process in your centre?

Question instructions: *Select all that apply*

- ☐ a) Assess transition readiness
- ☐ b) Identify patient needs
- ☐ c) Monitor progress
- ☐ d) Provide psychological support
- ☐ e) I do not use questionnaires in my clinical practice (please specify) ____________

10 Which e-health tools you currently use are most beneficial for supporting transition care?

Question instructions: *Select one answer*

- ○ a) Mobile applications for patient education and monitoring
- ○ b) Online platforms for tracking transition readiness and progress
- ○ c) Telehealth consultations to facilitate communication between paediatric and adult teams
- ○ d) Digital tools for patient self-management (e.g., reminders, trackers)
- ○ e) None / I do not currently use eHealth tools
- ○ f) Other (please specify) ____________

11 Which e-health tools do you think could be most beneficial for supporting transition care in the future?

Question instructions: *Select one answer*

- ○ a) Mobile applications for patient education and monitoring
- ○ b) Online platforms for tracking transition readiness and progress
- ○ c) Telehealth consultations to facilitate communication between paediatric and adult teams
- ○ d) Digital tools for patient self-management (e.g., reminders, trackers)
- ○ e) None
- ○ f) Other (please specify) ____________

12 What is the main barrier to implementing e-health tools for transition care in your centre?

Question instructions: *Select one answer*

- ○ a) Lack of resources or funding
- ○ b) Limited digital literacy among patients or caregivers
- ○ c) Resistance to change from healthcare providers
- ○ d) Absence of standardized e-health platforms or guidelines
- ○ e) Data security concerns (ensuring compliance with data protection regulations)
- ○ f) Other (please specify) [ ]

Caregiver Involvement

Map caregiver roles in the transition process and support offered to them.

13 What strategies would most enhance caregiver involvement and support during transition?

Question instructions: *Select one answer*

- ○ a) Joint training sessions for caregivers and healthcare teams on the transition process
- ○ b) Development of individualized care plans and clear guidelines on caregiver roles in the transition setting
- ○ c) Opportunities for caregivers to meet with adult medical team before the formal transition
- ○ d) Resources and support groups for caregivers to share experiences and strategies
- ○ Other (please specify) [ ]

14 What challenges do caregivers report during the transition?

Question instructions: *Select all that apply*

- ☐ a) Lack of information about adult care
- ☐ b) Limited communication between paediatric and adult teams
- ☐ c) Lack of support to help them manage their roles effectively
- ☐ d) Emotional stress and uncertainty regarding the process
- ☐ Other (please specify) [ ]

Timing and Criteria for Transition

Examine the criteria (e.g., age, clinical stability) for determining the ideal time to start the transition.

15 What age do you deem ideal to initiate the transition process?

Question instructions: *Select one answer*

- ◯ a) <14 years
- ◯ b) 14-16 years
- ◯ c) 16-18 years
- ◯ d) >18 years
- ◯ Other (please specify) [ ]

16 What criteria do you use (or consider appropriate) to determine the start of the transition process?

Question instructions: *Select all that apply*

- ☐ a) Patient age
- ☐ b) Clinical stability of the condition
- ☐ c) Patient's readiness and self-management skills
- ☐ d) Mental health status
- ☐ e) Recommendations or requirements from local or national protocols
- ☐ f) Patient's and/or parents' wish
- ☐ Other (please specify) [ ]

17 Which tool is typically used in your centre to assess the optimal timing for transition?

Question instructions: *Select one answer*

- ◯ a) Personal judgment of healthcare providers
- ◯ b) Multidisciplinary team decisions
- ◯ c) Standardized readiness assessment tools (e.g., TRAQ, Ready Steady Go)
- ◯ d) Local hospital or national protocols
- ◯ Other (please specify) [ ]

Collaboration Among Health Professionals

Analyse paediatric-adult physician collaboration and the transfer of clinical information.

18 Which of the following represents the main unmet need in the collaboration between paediatric and adult physicians during transition in your centre?

Question instructions: *Select one answer*

- ◯ a) Ensuring effective communication of patient medical history, treatment plans, and ongoing needs
- ◯ b) Co-creating individualized care plans that reflect the unique needs of transitioning patients
- ◯ c) Establishing standardized protocols for transferring clinical information
- ◯ Other (please specify) [ ]

19 What is the key barrier to seamless collaboration between paediatric and adult endocrinologists in your centre?

Question instructions: *Select one answer*

- ○ a) Lack of multidisciplinary team meetings
- ○ b) Insufficient shared outpatient clinics during the transition phase
- ○ c) Limited availability of transition-specific communication tools
- ○ d) Frequent changes in contact persons at the paediatric or adult centre
- ○ Other (please specify) [ ]

20 Which strategy could most enhance collaboration between paediatric and adult health professionals in your centre?

Question instructions: *Select one answer*

- ○ a) Organizing joint discussions using standardized checklists or transition flowcharts
- ○ b) Developing shared transition curricula for health professionals
- ○ c) Greater involvement of young patients in identifying gaps in transition care and co-designing new tools
- ○ d) Greater involvement of patient support groups in transition of care pathways
- ○ Other (please specify) [ ]

Psychological support

Evaluate the role and the involvement of clinical psychologists in assessing patients' needs and readiness for transition, helping develop transition plans, promote support and treatment adherence.

21 Is psychological readiness assessed during the transition process at your centre?

Question instructions: *Select one answer*

- ○ a) By psychologists specialized in the patient's specific condition
- ○ b) By general psychologists within the healthcare unit
- ○ c) Through external psychological services or consultants
- ○ d) Using standardized tools/questionnaires (e.g., TRAQ, Transition Readiness Assessment)
- ○ e) Psychological readiness is not formally assessed
- ○ Other (please specify) [ ]

22 Are psychologists involved in developing transition plans for patients at your centre?

Question instructions: *Select one answer*

- ◯ a) Yes, for all patients
- ◯ b) Yes, but only for patients with specific psychological needs
- ◯ c) Yes, but only for conditions where psychologists have expertise
- ◯ d) No, although psychologists are available, they are not involved in transition planning
- ◯ e) There is no access to psychologists at my centre

23 What role do psychologists play in post-transition follow-up care at your centre?

Question instructions: *Select one answer*

- ◯ a) They continue to provide care within the same healthcare unit.
- ◯ b) They oversee planned follow-up visits for a specific period post-transition.
- ◯ c) Responsibility is transferred to psychologists in the adult care unit.
- ◯ d) Psychological support is not provided after the transition.

Long-term Monitoring

Review follow-up protocols for post-transition care and continuity of care strategies in Endo-ERN centres.

24 How does your centre evaluate the effectiveness of long-term follow-up care post-transition?

Question instructions: *Select one answer*

- ◯ a) Regular patient satisfaction surveys
- ◯ b) Clinical outcome measures (e.g., hospitalization rates, treatment adherence)
- ◯ c) Feedback from multidisciplinary team meetings
- ◯ d) Communication with the adult centre's personnel
- ◯ e) We do not evaluate long-term follow-up effectiveness
- ◯ Other (please specify) ______

25 What specific challenges do you encounter in ensuring long-term follow-up for transitioned patients?

Question instructions: *Select one answer*

- ◯ a) Poor attendance at follow-up appointments
- ◯ b) Limited resources for ongoing monitoring
- ◯ c) Lack of dedicated transition coordinators
- ◯ d) Inconsistent communication between care teams
- ◯ e) I encounter no specific challenges
- ◯ Other (please specify) [ ]

26 What tools or strategies does your centre prioritize to enhance long-term follow-up care?

Question instructions: *Select one answer*

- ◯ a) Regular telehealth check-ins
- ◯ b) Long-term care plans co-developed by the paediatric and adult providers
- ◯ c) Multidisciplinary meetings to track patient progress
- ◯ d) Use of standardized health tracking tools (e.g., questionnaires, EHRs)
- ◯ e) None
- ◯ Other (please specify) [ ]

27 How satisfied are you with the current transition of care policies and procedures in place at your institution?

Question instructions: *Select one answer*

◯ a) Very dissatisfied ◯ b) Dissatisfied ◯ c) Neutral ◯ d) Satisfied ◯ e) Very satisfied

Equity of Access to Services

Explore disparities in access to transition services based on region, socio-economic status, and ethnicity.

28 In your country, is the quality of care during the transition process influenced by regional differences (e.g., geography, availability of specialized centres)?

Question instructions: *Select one answer*

◯ a) Strongly agree ◯ b) Agree ◯ c) Neutral ◯ d) Disagree ◯ e) Strongly disagree

29 Does socio-economic status influence transition of care in your country?

Question instructions: *Select one answer*

- ◯ a) Strongly agree
- ◯ b) Agree
- ◯ c) Neutral
- ◯ d) Disagree
- ◯ e) Strongly disagree

30 To what extent does ethnicity influence the quality of care provided during the transition process in your country?

Question instructions: *Select one answer*

- ◯ a) Ethnicity exerts a significant negative influence
- ◯ b) Ethnicity exerts a moderate negative influence
- ◯ c) Ethnicity can exert a negative influence, depending on the specific condition
- ◯ d) Ethnicity does not exert an influence on quality of care
- ◯ e) I am unsure of the impact

Patient Experience

Gather patient feedback on transition experiences, including satisfaction and challenges faced.

31 Do you systematically collect patient feedback on their transition experience?

Question instructions: *Select one answer*

- ◯ a) Yes, feedback is collected at multiple points during the transition
- ◯ b) Yes, feedback is collected at the end of the transition
- ◯ c) Yes, but only with regards to specific conditions
- ◯ d) No, patient feedback is not collected systematically

32 How is patient feedback on the transition process used in your centre?

Question instructions: *Select one answer*

- ◯ a) To improve existing transition protocols
- ◯ b) To identify gaps in the transition process
- ◯ c) To develop personalized care plans
- ◯ d) Feedback is not systematically collected or used
- ◯ Other (please specify) __________

33 What are the main challenges patients report during the transition?

Question instructions: *Select all that apply*

- ☐ a) Lack of clear communication between paediatric and adult care teams
- ☐ b) Insufficient preparation for adult care
- ☐ c) Limited psychological or emotional support
- ☐ d) Disparities in access to healthcare services
- ☐ e) Lack of a dedicated contact person at the adult care centre
- ☐ Other (please specify) ____

Privacy and Data Security

Investigate privacy practices and patient data management during the transition process.

34 Are patients asked for consent to medical data transfer during the transition?

Question instructions: *Select one answer*

- ○ a) Yes, consent is explicitly obtained for all data transfers
- ○ b) Yes, but it is implicit in accepting patient transfer
- ○ c) No, consent is not routinely obtained
- ○ d) I am not aware of the consent procedures at my centre

35 How is the patient's medical history transferred during the transition?

Question instructions: *Select one answer*

- ○ a) Digitally, through Electronic Health Records (EHRs) or secure platforms
- ○ b) Paper-based records only
- ○ c) A combination of digital and paper records
- ○ Other (please specify) ____

36 Additional comments or observations?

Question instructions: *Feel free to share additional information regarding your answers, or concerning 'transition of care' details or procedures at your centre.*
