# Supplementary material for: Mapping transition of care for rare endocrine conditions: findings from a cross-sectional survey by the Endo-ERN ToC Working Group
Source: Endocr Connect. 2026 Apr 15;15(4):e250798. doi: 10.1530/EC-25-0798 (PMC13097265; doi:10.1530/EC-25-0798)
Supplement: Supplementary file 2 [file supplementary_table.pdf]

Country (Total 21)	Centre name (total 80)	ERN (Total 65)	Responses (Total 111)
**Austria**	Klinikum Wels-Grieskirchen,Center for Pediatric Endocrinology / Dpt. of Pediatrics	Yes	1
	Medical University of Vienna	Yes	1
**Belgium**	AZ Sint-Jan Bruges, Belgium	No	1
	Ghent University Hospital	Yes	1
	Hôpital Universitaire de Bruxelles (HUDERF-Erasme)	Yes	3
	UZ Antwerpen	Yes	1
	UZ Brussels	Yes	1
	UZ Leuven	Yes	0
	Cliniques universitaires Saint Luc	Yes	0
	Centre Hospitalier Universitaire de Liège	Yes	0
**Bulgaria**	Clinic of Genetics, SBALDB "Prof Ivan Mitev"	No	1
	Medical University -Sofia, Faculty of Medicine, University Pediatric Hospital "Prof. Ivan Mitev" Sofia	No	1
	UMHAT "Sveta Marina" (Varna)	Yes	3
	University Hospital "St. George", Plovdiv. Bulgaria	No	1
	USHATE "ACAD IVAN Penchev"	Yes	1
**Croatia**	Vuk Vrhovac University Clinic for Diabetes, Endocrinology and Metabolic Diseases, Merkur University Hospital	Yes	0
	University Hospital Centre Sestre Milosrdnice University Department of Oncology & Nuclear Medicine Centre of Reference for thyroid disease	Yes	0
**Cyprus**	Department of Molecular Genetics, Function and Therapy, The Cyprus Institute of Neurology and Genetics1.	Yes	2
**Czech Republic**	General University Hospital in Prague	Yes	1
	University Hospital Motol	Yes	1
**Denmark**	Aarhus University Hospital	Yes	6
	Childrens departament Regionshospital i Nordjylland Hjørring	No	1
	RH Gødstrup, Herning, Denmark	No	1
	Odense Universitetshospital	Yes	0
	Copenhagen University Hospital, Rigshospitalet	Yes	0
**Estonia**	Tartu University Hospital	Yes	0
**Finland**	Helsinki University Hospital, Hospital District of Helsinki and Uusimaa	Yes	1
**France**	Assistance Publique – Consortium Cochin, Robert Debré, Necker, St Antoine, La Pitié Salpétrière, Trousseau University Hospitals	Yes	1
	Assistance Publique – Consortium Pitie Salpêtrière – French Reference Centre for Prader-Willi Syndrome	Yes	1
	Assistance Publique – Hôpitaux de Marseille	Yes	1
	Hospices Civils de Lyon	Yes	1
	CHU Toulouse	Yes	1

	Reference centre for rare diseases of calcium and phosphate-HEGP	Yes	0
	Hôpital Bicêtre	Yes	0
	CHU Angers	Yes	0
**Germany**	Charité Universitätsmedizin Berlin	Yes	2
	Department of women's health, pediatric and adolescent gynecology, Women's university hospital, Tuebingen, Germany	No	1
	Hannoversche Kinderheilanstalt, Diabetes Centre for Children and Adolescents	Yes	1
	Katholisches Klinikum Bochum	Yes	1
	Ludwig-Maximilian-University Munich	Yes	1
	Poliklinik am HELIOS-Klinikum Berlin-Buch, Germany	No	1
	Universitätsklinikum Schleswig-Holstein	Yes	2
	Universitätsklinikum Tübingen	Yes	1
	Universitätsklinikum Ulm (Ulm University Medical Center)	Yes	1
	University Hospital Essen – Center for Rare Endocrine Diseases	Yes	1
	University Medical Center Mainz	Yes	1
	University Hospital Würzburg	Yes	0h
	University Hospital Aachen	Yes	0
	Universitaetsklinikum Duesseldorf, AöR, Klinik für Allgemeine Pädiatrie, Neonatologie und Kinderkardiologie	Yes	0
	Otto-von-Guericke University, Med. Faculty – Central German Network for Rare diseases	Yes	0
	Charité Universitätsmedizin Berlin	Yes	0
**Greece**	Aghia Sophia Children’s Hospital	Yes	1
	Evangelismos General Hospital	Yes	1
	General Hospital of Athens “LAIKO”	Yes	2
**Hungary**	Semmelweis University	Yes	0
**Ireland**	Beaumont Hospital & Children’s Health Ireland	Yes	1
**Italy**	Antonio Cardarelli Hospital- Azienda Ospedaliera di rilievo nazionale	Yes	1
	AOU policlinico “G.Martino” Messina	Yes	1
	AOU Università degli Studi della Campania “Luigi Vanvitelli”, Napoli	Yes	1
	AOUI di Verona	Yes	1
	Azienda Ospedaliera di Padova (AOP)	Yes	1
	Azienda Ospedaliero Universitaria Città della Salute e della Scienza di Torino	Yes	4
	Azienda Ospedaliero Universitaria di Ferrara	Yes	1
	Azienda Ospedaliero-Universitaria Policlinico Umberto I	Yes	1
	Azienda Unità Sanitaria Locale-IRCCS of Reggio Emilia	Yes	1
	Bambino Gesu' Children Hospital, Rome, Italy	No	3

	Fondazione IRCCS Ca' Granda Ospedale Maggiore Policlinico, Milano	Yes	2
	IRCCS Azienda Ospedaliero-Universitaria di Bologna	Yes	2
	IRCCS Istituto Giannina Gaslini, Genova	Yes	1
	Istituto Auxologico Italiano – Istituto di Ricovero e Cura a Carattere Scientifico	Yes	1
	Obesity and Lipodystrophy Centre, Endocrinology Unit, Azienda Ospedaliero Universitaria Pisana	Yes	1
	Meyer Children's Hospital Florence	Yes	1
	Ospedale San Raffaele	Yes	3
	University Hospital Florence	Yes	2
	IRCCS Ospedale Policlinico San Martino – Genova – Italy	Yes	0
	AOU di Modena	Yes	0
**Latvia**	Pauls Stradins Clinical University Hospital	Yes	0
	Childrens Clinical University Hospital	Yes	0
**Lithuania**	Hospital of Lithuanian University of Health Sciences Kauno Klinikos	Yes	2
**Luxembourg**	Centre Hospitalier de Luxembourg	Yes	0
**Malta**	Mater Dei Hospital	Yes	0
**Norway**	Oslo University Hospital HF	Yes	0
	Helse Bergen HF, Haukeland University Hospital	Yes	0
**Poland**	Karol Jonscher's Clinical Hospital of Poznan University of Medical Sciences	Yes	1
	Maria Sklodowska-Curie National Research Institute of Oncology (MSCNRIO)	Yes	1
	Public Pediatric Teaching Hospital	Yes	1
	Saint John Paul II Upper Silesian Child Health Centre, Public Clinical Hospital no.6 of the Medical University of Silesia in Katowice	Yes	0
**Portugal**	Unidade Local de Saúde de Santa Maria(ULS de Santa Maria)	Yes	0
	Centro Hospitalar Universitario de São João, EPE	Yes	0
	APDP – Diabetes Portugal	Yes	0
**Romania**	Institute of Oncology "Prof dr. Ion Chiricuta" CLUJ NAPOCA	Yes	1
	Spitalul Clinic de Psihiatrie "Prof. dr. Alexandru Obregia"	Yes	1
	Spitalul Clinic Judetean de Urgenta Cluj (Cluj County Emergency Clinical Hospital)	Yes	0
	Spitalul Clinic de Urgenta pentru Copii, Cluj-Napoca	Yes	0
**Slovakia**	University Hospital Martin, Dept of Paediatrics of Jessenius Medical Faculty Comenius University	Yes	1
	National Institute of Children's Diseases, Dept. of Paediatrics of Medical Faculty Comenius University	Yes	0
**Slovenia**	University Medical Centre Ljubljana	Yes	1
**Spain**	Fundacio de Gestio Sanitaria Hospital de la Santa Creu i Sant Pau	Yes	1

	Hospital Universitari Vall d’Hebron	Yes	1
	Hospital Universitario Cruces	Yes	0
	Hospital Sant Joan de Déu Barcelona	Yes	0
	Complejo Hospitalario Regional Virgen del Rocío	Yes	0
**Sweden**	Karolinska University Hospital	Yes	3
	Sahlgrenska University Hospital	Yes	1
**The Netherlands**	Erasmus MC: University Medical Center Rotterdam	Yes	1
	Franciscus Rotterdam en Schiedam, Netherlands	No	1
	Leiden University Medical Center	Yes	5
	Máxima Medisch Centrum	Yes	1
	Radboud University Nijmegen Medical Center – including Amalia’s children Hospital	Yes	4
	Reinier de Graaf Groep Delft The Netherlands	No	1
	Rijnstate Hospital	No	1
	University Medical Center Groningen	Yes	1
	University Medical Center Utrecht – Dept of Endocrine Oncology	Yes	1
	Maastricht University Medical Center – Center for Thyroid Carcinoma	Yes	0
	Amsterdam UMC	Yes	0
**United Kingdom**	Royal Hospital for Children, Glasgow, UK	No	1

Supplementary Table 1. *Participating and non-participating centres, divided by country and by Endo-ERN status, alongside number of responses. Note: non-participating European countries include Croatia, Estonia, Hungary, Latvia, Luxembourg, Malta, Norway and Portugal.*
